# Antihyperglycemic effects of *Lysiphyllum strychnifolium* leaf extract *in vitro* and *in vivo*

**DOI:** 10.1080/13880209.2022.2160771

**Published:** 2023-01-10

**Authors:** Arman Syah Goli, Vilasinee Hirunpanich Sato, Hitoshi Sato, Savita Chewchinda, Jiraporn Leanpolchareanchai, Jannarin Nontakham, Jantana Yahuafai, Thavaree Thilavech, Pongsatorn Meesawatsom, Metawee Maitree

**Affiliations:** a Department of Pharmacology, Faculty of Pharmacy, Mahidol University, Thailand; b Center of Biopharmaceutical Science for Healthy Ageing, Faculty of Pharmacy, Mahidol University, Bangkok, Thailand; c Division of Pharmacokinetics and Pharmacodynamics, Department of Pharmacology, Toxicology and Therapeutics, School of Pharmacy, Showa University, Japan; d Department of Food Chemistry, Faculty of Pharmacy, Mahidol University, Thailand; e Department of Pharmacy, Faculty of Pharmacy, Mahidol University, Thailand; f Clinical Research Section, Division of Research and Academic Support, National Cancer Institute, Bangkok, Thailand

**Keywords:** α-glucosidase, glucose uptake, GLUT4, oral glucose tolerance test

## Abstract

**Context:**

*Lysiphyllum strychnifolium* (Craib) A. Schmitz (LS) (Fabaceae) has traditionally been used to treat diabetes mellitus.

**Objective:**

This study demonstrates the antidiabetic and antioxidant effects of aqueous extract of LS leaves *in vivo* and *in vitro*.

**Materials and methods:**

The effects of aqueous LS leaf extract on glucose uptake, sodium-dependent glucose cotransporter 1 (SGLT1) and glucose transporter 2 (GLUT2) mRNA expression in Caco-2 cells, α-glucosidase, and lipid peroxidation were evaluated *in vitro*. The antidiabetic effects were evaluated using an oral glucose tolerance test (OGTT) and a 28-day consecutive administration to streptozotocin (STZ)-nicotinamide (NA)-induced type 2 diabetic mice.

**Results:**

The extract significantly inhibited glucose uptake (IC_50_: 236.2 ± 36.05 µg/mL) and downregulated SGLT1 and GLUT2 mRNA expression by approximately 90% in Caco-2 cells. Furthermore, it non-competitively inhibited α-glucosidase in a concentration-dependent manner with the IC_50_ and *K_i_* of 6.52 ± 0.42 and 1.32 µg/mL, respectively. The extract at 1000 mg/kg significantly reduced fasting blood glucose levels in both the OGTT and 28-day consecutive administration models as compared with untreated STZ-NA-induced diabetic mice (*p* < 0.05). Significant improvements of serum insulin, homeostasis model assessment of insulin resistance (HOMA-IR), and GLUT4 levels were observed. Furthermore, the extract markedly decreased oxidative stress markers by 37–53% reduction of superoxide dismutase 1 (SOD1) in muscle and malondialdehyde (MDA) in muscle and pancreas, which correlated with the reduction of MDA production *in vitro* (IC_50_: 24.80 ± 7.24 µg/mL).

**Conclusion:**

The LS extract has potent antihyperglycemic activity to be used as alternative medicine to treat diabetes mellitus.

## Introduction

Diabetes mellitus (DM) is a chronic multi-system disease associated with either abnormality of insulin production in the pancreas, incapability of insulin utilization or both (American Diabetes Association [ADA] 2020). Currently, many synthetic drugs for the management of DM are available in the pharmaceutical market. Despite the advantageous effects of these drugs to treat type 2 diabetes mellitus (T2DM), there are unavoidable adverse effects, including hypoglycemia, gastrointestinal disorders, weight gain, lactic acidosis, bone fracture, peripheral edema, and cardiovascular disorders (Zhou et al. 2016). Moreover, the medicinal cost of diabetes treatment has become an enormous burden to societies, attracting the attention of many researchers to develop new antidiabetic medicines or supplements from herbs with higher safety.

*Lysiphyllum strychnifolium* (Craib) A. Schmitz (Fabaceae) (synonym; *Bauhinia strychnifolia*) has been widely used as a traditional Thai medicine for alcohol detoxification (Hao et al. 2003; Luengthong et al. 2016). The active compounds discovered from the leaves of *Lysiphyllum strychnifolium* (LS) include gallic acid, trilobatin and yanangdaengin (Kongkiatpaiboon et al. 2020). Various biological properties of LS extracts, comprising anti-cancer, antiallergic, antimicrobial, anti-inflammatory, anti-hyperuricemic, and antidote effects, have been reported (Yuenyongsawad et al. 2013; Bunluepuech et al. 2013; Sato et al. 2019; Sukprasert, Deenonpoe et al. 2020; Sukprasert, Pansuksan et al. 2020). Recently, some isolated compounds from LS stem, such as 3,5,7-trihydroxychromone-3-*O*-α-l-rhamnopyranoside and 3,5,7,3′,5′-pentahydroxyflavanonol-3-*O*-α-l-rhamnopyranoside, have been reported to exhibit antidiabetic activity by inhibiting intestinal hydrolyzing enzymes (α-amylase and α-glucosidase) and downregulating the mRNA expression of intestinal glucose transporters, sodium-dependent glucose cotransporters 1 (SGLT1) and glucose transporter 2 (GLUT2), in Caco-2 cells (Bunluepuech et al. 2019; Noonong et al. 2022). Moreover, evidence indicates that resveratrol, epicatechin, quercetin, and gallic acid isolated from LS stems inhibited α-glucosidase and DPP-IV enzymes, and quercetin promoted glucose storage in 3T3-L1 adipocyte cells (Praparatana et al. 2022). Additionally, the antioxidant capacity of LS extracts might be hypothesized as one of its antidiabetic mechanisms (Maitree et al. 2018; Sato et al. 2019; Kongkiatpaiboon et al. 2020), as oxidative stress is closely associated with the etiology of T2DM (Burgos-Moron et al. 2019). However, the pharmacological evidence on the antidiabetic effect of LS extracts has been limited to *in vitro* experimental systems, using only its stem part, while its antioxidant effect still focused on the reactive oxygen species (ROS) inhibition steps at the early state of lipid peroxidation.

No published literature has been found reporting the inhibitory effect of the aqueous extract of LS leaves on α-glucosidase enzyme, glucose uptake and glucose transporters in Caco-2 cells, lipid peroxidation *in vitro*, as well as its antidiabetic activity *in vivo*. Therefore, the present study aimed to elucidate the mechanisms underlying the antihyperglycemic and antioxidant activities of LS leaf extract in pharmacological and biochemical basis under *in vitro* and *in vivo* conditions. The inhibitory effects of LS leaf extract on glucose uptake and the gene expression of glucose transporters in Caco-2 cells, and α-glucosidase were carried out *in vitro*. The hypoglycemic effects were examined in the streptozotocin (STZ)-nicotinamide (NA)-induced T2DM mice model. Furthermore, the oxidative stress parameters *in vivo* and malondialdehyde (MDA) production as final stage of lipid peroxidation *in vitro*, were also evaluated.

## Materials and methods

### Chemicals and reagents

Acarbose, α-glucosidase and nicotinamide were purchased from Sigma-Aldrich Chemical Co. (St. Louis, MO, USA). Streptozotocin (STZ) was obtained from Merck KGaA (Darmstadt, Germany). All other reagents were of analytical grade and used without further purification.

### Identification of plant materials

The leaves of LS were collected at Ratchaburana, Bangkok, Thailand in June, 2020 and authenticated by Department of Pharmaceutical Botany, Faculty of Pharmacy, Mahidol University, Bangkok, Thailand, where the voucher specimen (voucher no. PBM-005667) was deposited.

### Preparation of the LS leaf extract

The LS leaf extract was prepared as described previously (Sato et al. 2019). The finely grinded leaves were extracted in boiling water with the water-to-leaf ratio of 10:1 (in mL/g) for 15 min in triplicate. The supernatant solution was filtered and subsequently concentrated using a controlled-vacuum rotary evaporator (Büchi, Flawil, Switzerland) at 60 °C and further lyophilized by a freeze dryer (Labconco, MO). The extract was preserved in −20 °C until used. The percentage yield was calculated by the following equation (Sato et al. 2019):

(1) $$\text{The percentage yield }(\%)\;=\frac{\text{weight of the extract}}{\text{weight of the powdered leaves}}\times 100$$

### Quantification of phytochemical compounds by high-performance liquid chromatography (HPLC)

The biomarkers, i.e., gallic acid, trilobatin and yanangdaengin in the LS extract were identified using the HPLC method based on a previous study (Kongkiatpaiboon et al. 2020). The Thermo HPLC system (Thermo Separation Products Inc., Waltham, MA) consisted of a vacuum solvent degasser, quaternary pump, autosampler, UV-visible diode-array detector, and a BDS Hypersil C18 column (150 mm × 4.6 mm, i.d. 5 μm) (Thermo Fisher Scientific Inc., Waltham, MA) was employed to determine the biomarkers in the LS extract. The standards of 1–32 μg/mL gallic acid, 31–1000 μg/mL trilobatin, and 15–500 μg/mL yanangdaengin dissolved in methanol were used for calibrations. A portion of the extract (5 mg) was dissolved in 1 mL methanol and sonicated for 20 min. The resultant solution was filtered through a 0.45 µm nylon membrane and 10 μL of the filtrate was injected into the HPLC system for analysis. The chromatographic separation was carried out using a mobile phase of 0.5% acetic acid as solvent A and methanol as solvent B at the flow rate of 1 mL/min at 25 °C. The elution gradient program employed was as follows: 0–100% B (40 min), 100% B (10 min), and subsequent column equilibration with 100% A for 10 min. The eluted peaks were detected at 254 nm and identified by comparing the retention time with standards. The calibration equations of *y* = 17532*x* − 9247.3 (*r*^2^ = 0.9909), *y* = 2519.7*x* − 20599 (*r*^2^ = 0.9995), and *y* = 7058.1*x* − 43413 (*r*^2^ = 0.9978) were used to quantify the contents of gallic acid, trilobatin, and yanangdaengin, respectively, in the LS extract.

### In vitro *antihyperglycemic effect of the LS extract*

#### Caco-2 cells culture and cell viability assay

The Caco-2 (ATCC^®^ HTB-37™) human intestinal cell monolayers were incubated at 37 °C in humidified incubator with 95% air/5% CO_2_ and maintained in complete medium containing dulbecco’s modified eagle medium (DMEM) with 25 mM glucose, 10% fetal bovine serum (FBS) and 1% penicillin/streptomycin. The cells were sub-cultured when reaching more than 80% confluency. For glucose uptake and gene expression analysis, Caco-2 cells were seeded into 12-well plates at a density of 6 × 10^4^ cells/well and cultured for 21 days with medium renewal every 2 days (Hubatsch et al. 2007).

The 3-(4,5-dimethylthiazol-2-yl)-2,5-diphenyl-2H-tetrazolium bromide (MTT) assay was employed to determine the percentage viability of cells upon treatment with extract (125–1000 μg/mL) using the method described by the previous study (Abu Bakar et al. 2020). The percentage of cell viability was calculated by the following equation (Abu Bakar et al. 2020):

(2) $$\text{Cell viability }(\%)=\frac{\text{Absorbance of treated well}}{\text{Absorbance of control well}}\times 100$$

#### Effect on glucose uptake in Caco-2 cells

The cultured Caco-2 cells were incubated with glucose-free DMEM for 2 h and subsequent incubation for 30 min with Hanks’ balanced salt solution (HBSS), pH 7.4, prior to glucose uptake experiment. The cells were initially incubated with 0.4 mL HBSS containing the LS extract (125–500 μg/mL) or phloridzin (10 μg/mL), as a positive control, for 10 min and further replenished with 1.6 mL glucose (0.55 mM) with 40 min incubation. The glucose content in cells was determined using a glucose assay kit (Abcam, Cambridge, UK), whereas the protein content was quantified using Bradford’s method (Sigma-Aldrich, St. Louis, MO) (Zhang et al. 2015; Li et al. 2018).

#### SGLT1 and GLUT2 mRNA expression in Caco-2 cells

The Caco-2 cells were incubated with serum and glucose-free DMEM for 2 h and subsequent incubation with the LS extract (125–250 µg/mL) in serum-free medium for 16 h (Alzaid et al. 2013). Then, total RNA from Caco-2 cells was extracted using RiboZol reagent (Solon, OH, USA) and the cDNA was synthesized using an ImProm-II^TM^ Reverse Transcription System kit (Promega, VIC, Australia) based on the manufacturer’s instructions. SGLT1, GLUT2 and glyceraldehyde-3-phosphate dehydrogenase (GAPDH) mRNA expression were analyzed by CFX96^TM^ Real-Time PCR (Bio-Rad Laboratories, Inc., Hercules, USA) using SsoFastTM EvaGreen^®^ Supermix kit. Thermocycling conditions were set at an annealing temperature of 59.5 °C at 40 PCR cycles for GAPDH and at an annealing temperature of 56.4 °C for 60 PCR cycles for SGLT1 and GLUT2. Quantitative measurements of glucose transporter relative to GAPDH gene expression were derived using the 2^−ΔΔ^*^CT^* method. The data were expressed as the relative to untreated control group.

#### α-Glucosidase activity

The α-glucosidase inhibitory activity was determined by a spectrophotometric method according to the previous study (Chewchinda et al. 2021). The percentage of inhibition was calculated by the following equation (Chewchinda et al. 2021):

(3) $$\text{α-Glucosidase inhibitory activity}=\;[\frac{A_{c}-A_{s}}{A_{c}}]\times 100$$

where *A_c_* and *A_s_* are the absorbance of control and sample, respectively.

The IC_50_ value was calculated from the plots of percentage inhibition of α-glucosidase activity versus log-concentration of the LS extract. Moreover, to further determine the type of enzyme inhibition of the LS extract on α-glucosidase activity, Lineweaver-Burk plot analysis was performed. The Michaelis-Menten constant (*K_m_*), the inhibition constant (*K_i_*) and maximum metabolic rate (*V*_max_) were examined by a non-linear regression analysis using Solver add-in equipped with Microsoft Excel 2010, by the following equation (Chewchinda et al. 2021):

(4) $$\nu =\;\frac{V_{\max\;\cdot }S}{K_{m}\;+S\;[1\;+\;\frac{I}{K_{i}}]}$$

where *v* represents the reaction velocity (mmol/min), *S* and *I* represent the substrate and inhibitor concentrations in the unit of µM and µg/mL, respectively.

### In vivo *antihyperglycemic activity of the LS extract*

#### Animals

Male ICR mice (30–40 g) were housed in the Animal Center at Faculty of Pharmacy, Mahidol University at a constant temperature (25 °C) with a 12 h light-dark cycle and had free access to standard diet and water *ad libitum*. The experimental protocol was approved by the Animal Ethics Committee of Faculty of Pharmacy, Mahidol University (permission number: PYT 008/2560 and PYT004/2020).

#### Induction of type 2 diabetes in mice

After 15 min a single dose i.p. administration of 120 mg/kg nicotinamide (NA) dissolved in normal saline, overnight-fasted mice (12 h) were intraperitoneally injected with 180 mg/kg STZ dissolved in 0.1 M citrate buffer (pH 4.5). After 14–21 days, the progression of diabetes was assessed by measuring blood glucose after overnight fasting. Blood was withdrawn from mouse’s tail vein and glucose level was measured by using a glucometer with glucose test strips (Accu-Chek^®^, Roche, Thailand). Mice with fasting blood glucose (FBG) exceeding 200 mg/dL were selected as STZ-NA-induced DM mice and further employed in the study (Chewchinda et al. 2021).

#### Oral glucose tolerance test (OGTT) in normal and diabetic mice

Hypoglycemic effect of the LS extract was evaluated by OGTT in both normal and STZ-NA-induced DM mice. The acute toxicity study by Chutoam et al. (2015) reported that signs and symptoms of toxicity were not found in mice administered with the aqueous extract of LS leaves at the doses up to 6 g/kg. Furthermore, our preliminary study found effective dose in the range of 500–1000 mg/kg. Hence, the favorable doses (500 and 1000 mg/kg) of LS extract were selected in this present study. To examine whether the LS extract produces a hypoglycemic effect in normal mice, the prepared doses of extract dissolved in water were orally administered in mice fasted for 12 h at 30 min before oral glucose loading (2 g/kg).

In a separate study, overnight fasting STZ-NA-induced type 2 diabetic mice were divided into four groups. They were orally administered with water, glibenclamide (5 mg/kg), as a positive control, and 500 and 1000 mg/kg of the LS extract, respectively, at 30 min before administration of glucose solution (2 g/kg).

Then, blood glucose was measured from tail vein at baseline (0), 30, 60, 90 and 120 min using a glucometer with glucose test strips (Accu-Chek^®^, Roche, Thailand). Area under the curve (AUC) of FBG concentrations over time (0–120 min) was calculated by a linear trapezoidal rule method as follows (Chewchinda et al. 2021):

(5) $${\mathrm{AUC}}_{0-120}=\frac{\left[C_{0}+C_{30}]\cdot [t_{30}{-t}_{0}\right]}{2}+\frac{\left[C_{30}+C_{60}]\cdot [t_{60}-t_{30}\right]}{2}+\frac{\left[C_{60}+C_{90}]\cdot [t_{90}-t_{60}\right]}{2}+\frac{\left[C_{90}+C_{120}]\cdot [t_{120}-t_{90}\right]}{2}$$

where *C_0_*, *C_30_*, *C_60_*, *C_90_* and *C_120_* are the FBG concentrations (mg/dL) at 0, 30, 60, 90 and 120 min after glucose administration, respectively, and *t_0_*, *t_30_*, *t_60_*, *t_90_* and *t_120_* are the times (min) after glucose administration, respectively.

### Hypoglycemic effect in STZ-NA-induced diabetic mice after continuous administration of the LS extract

#### FBG concentrations

The mice were assigned into five groups (*n* = 6–8). Group 1 (normal mice) and 2 (DM mice) were administered with water and nominated as normal control and STZ-NA-induced DM control mice, respectively. Group 3 was STZ-NA-induced DM mice receiving 5 mg/kg glibenclamide, as a positive control group. Group 4 and 5 were STZ-NA-induced DM mice treated with 500 and 1000 mg/kg of the LS extract, respectively. All treatments were administered *via* oral gavage once daily for 28 days.

During 28-days of treatment, body weight and food intake were daily measured. FBG was measured after 12 h fasting at day 0, 14 and 28 of the treatment periods. At the end of the study, mice were sacrificed using overdosed CO_2_ inhalation. Blood was collected *via* cardiac puncture and then centrifuged at 5000 rpm in 4 °C for 8 min to get serum. Furthermore, pancreas and gastrocnemius muscle were immediately harvested and rinsed two to three times in ice cold normal saline. The collected organs were minced into small pieces and homogenized in an ice-cold PBS (0.01 M, pH 7.4). The obtained homogenates were centrifuged at 5000 rpm for 15 min in 4 °C, then the supernatant was collected and used for the subsequent studies. The total protein content of homogenates was determined by Bradford protein assay kit (Sigma-Aldrich, St. Louis, MO).

#### Serum insulin levels and homeostasis model assessment of insulin resistance (HOMA-IR)

Serum insulin level was measured using the mouse insulin enzyme-linked immunosorbent assay (ELISA) kit (Fujifilm Wako, Osaka, Japan). All procedures were performed according to manufacturer’s guidelines. Then, data of FBG and serum insulin levels on day 28 were used to calculate HOMA-IR with the formula stated as follows (Onishi et al. 2010):

(6) $$\text{HOMA-IR}=\frac{\text{fasting insulin }(\mu \mathrm{IU}/\mathrm{mL})\times \text{fasting glucose }(\mathrm{mg}/\mathrm{dL})}{405}$$

#### GLUT4 expression in muscle

The muscle homogenates were used to quantify the protein expression of GLUT4 using mouse GLUT4 ELISA kit based on the protocol of manufacturer (Aviva Systems Biology Corporation, San Diego, CA). The concentration of GLUT4 expression in muscle was expressed as ng/mg protein.

### Measurement of oxidative stress markers

#### *Malondialdehyde (MDA) production* in vitro

The amount of produced MDA, a major byproduct of lipid peroxidation during oxidative stress, was measured according to the previous method with slight modification (Kikuzaki and Nakatani 1993). A mixture of 1 mL LS extract (7–125 μg/mL) or Trolox (3–469 ng/mL) as a positive control, 99.5% ethanol (4 mL), 2.5% linoleic acid in 99.5% ethanol (4.1 mL), 0.02 M phosphate buffer (pH 7.0, 8.0 mL) and 3.9 mL distilled water were reacted. The solution mixture was kept in the dark at 40 °C of incubator for 10 days. A mixture of 20% trichloroacetic acid (TCA) (2 mL) and 0.67% thiobarbituric acid (TBA) (2 mL) were added into 1 mL of solution mixture. The mixture resulted was boiled (95–100 °C) for 10 min and promptly cooled down under running tap water. Then, the mixture was centrifuged at 5000 rpm for 5 min and the absorbance was measured at 532 nm. The percentage of inhibition was calculated using the following formula (Kikuzaki and Nakatani 1993):

(7) $$\text{MDA production inhibitory activity}=\;[\frac{A_{c}-A_{s}}{A_{c}}]\times 100$$

where *A_c_* and *A_s_* are the absorbance of control and sample, respectively.

The MDA resulted was quantified as MDA equivalent (mg MDA/g linoleic acid) using the standard curve constructed by plotting the concentration of TMP (0.2–10.0 μg/mL) as MDA precursor against its absorbance and the IC_50_ value was calculated from the plots of percentage inhibition of MDA production versus concentration of the LS extract.

#### MDA in muscle and pancreas homogenates

MDA was measured in pancreas and muscle homogenates as previously described with few modifications (Gönenç et al. 2006; Demirbilek et al. 2020). For pancreas, the homogenates (300 μL) were deproteinized with 40% TCA (500 μL) and mixed with 0.67% of TBA (200 μL). Meanwhile in muscle, the homogenates were reacted with 40%TCA (600 μL) and 0.67% of TBA (600 μL). The solution mixture was heated at 95–100 °C for 30 min following cooled down using running tap water. The mixture was centrifuged at 5000 rpm for 15 min at 4 °C and the absorbance of supernatant was measured at 532 nm. The MDA content was quantified using TMP to construct the standard curve of MDA. The concentration of MDA was expressed as ng/mg protein.

#### Superoxide dismutase 1 (SOD1) in muscle homogenates

The amount of SOD1, as the endogenous antioxidant enzyme, was assessed in muscle homogenates using a commercial mouse SOD1 ELISA kit following the manufacturer’s instructions (Elabscience Biotechnology Inc., Houston, TX). The concentration of SOD1 was expressed as ng/mg protein.

#### Statistical analysis

Data are expressed as the mean ± standard error of the mean (SEM). Significant difference was analyzed using one-way ANOVA in SPSS program version 18 followed by *post hoc* Tukey’s multiple comparison. Correlation analysis between lipid peroxidation versus insulin and GLUT4 levels was performed using Pearson’s correlation coefficient test. The difference was statistically identified as significant if the probability value was less than 0.05 (*p* < 0.05).

## Results

### Plant extraction and quantitative phytochemical analysis by HPLC

**Figure 1.**
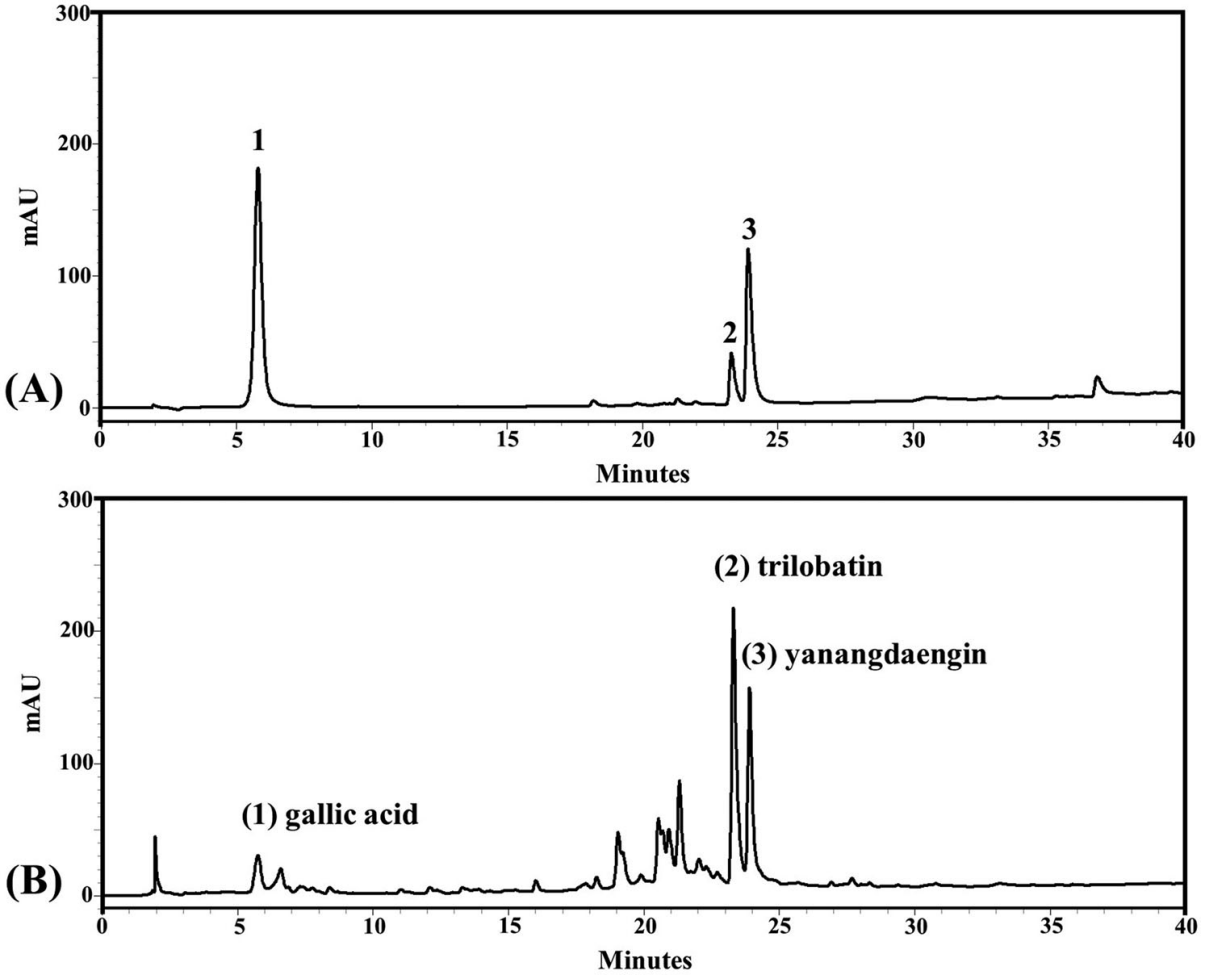
HPLC chromatograms of reference standards: gallic acid (1), trilobatin (2) and yanangdaengin (3), respectively [A] and the LS extract [B].

The physical appearance of LS leaf extract was dried powder with brownish color. The percent yield was 24.50% w/w. The HPLC chromatogram of the LS extract showed prominent peaks with the retention times of 5.8, 23.3 and 23.9 min which correspond to reference standards of gallic acid, trilobatin and yanangdaengin, respectively (Figure 1). The concentrations of gallic acid, trilobatin and yanangdaengin in the LS extract were calculated to be 0.39 ± 0.01, 15.08 ± 0.09 and 3.28 ± 0.05% w/w, respectively.

### In vitro *hypoglycemic effects of the LS extract*

#### Glucose uptake in Caco-2 cells

**Figure 2.**
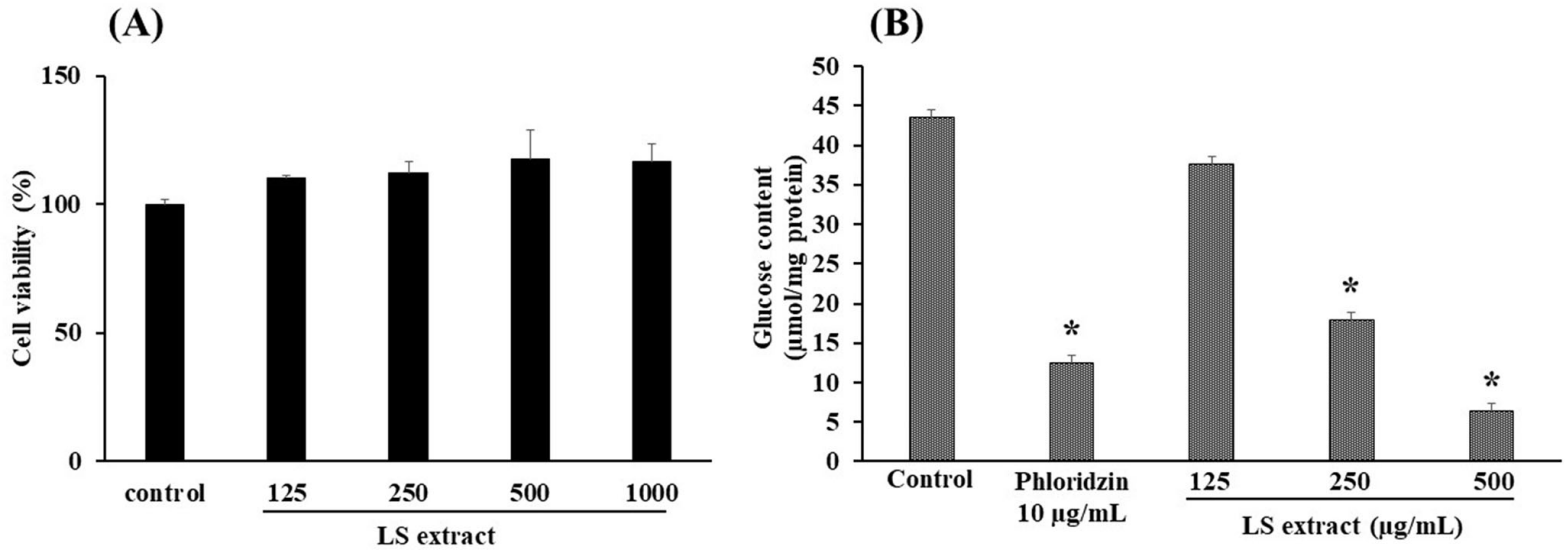
Effect of the LS extract on cell viability (A) and glucose uptake (B) in Caco-2 cells. Data are expressed as mean ± *SD* (*n* = 3). *Significant difference compared to control (*p* < 0.05).

After the treatment of Caco-2 cells with the LS extract at the concentrations ranged 125–1000 µg/mL for 24 h, the percentage of viable cells in the extract-treated groups was calculated and compared to the number of surviving cells in the control group (vehicle only). As shown in Figure 2(A), the LS extract did not affect the cell viability at the concentrations of 125–1000 µg/mL, indicating no toxicity on Caco-2 cells. Therefore, the concentration range of 125–500 µg/mL LS extract was selected for further determination of glucose uptake and gene expression of glucose transporters in Caco-2 cells in subsequent experiments.

As shown in Figure 2(B), the glucose uptake in Caco-2 cells with the presence of phloridzin (10 µg/mL), as a positive control, was significantly decreased by approximately 71%, as compared with control (*p* < 0.05). Incubation with the LS extract significantly inhibited the glucose uptake in Caco-2 cells in a concentration-dependent manner with the IC_50_ value of 236.2 ± 36.1 µg/mL, with statistical differences among the three concentrations employed.

#### SGLT1 and GLUT2 mRNA expressions in Caco-2 cells

**Figure 3.**
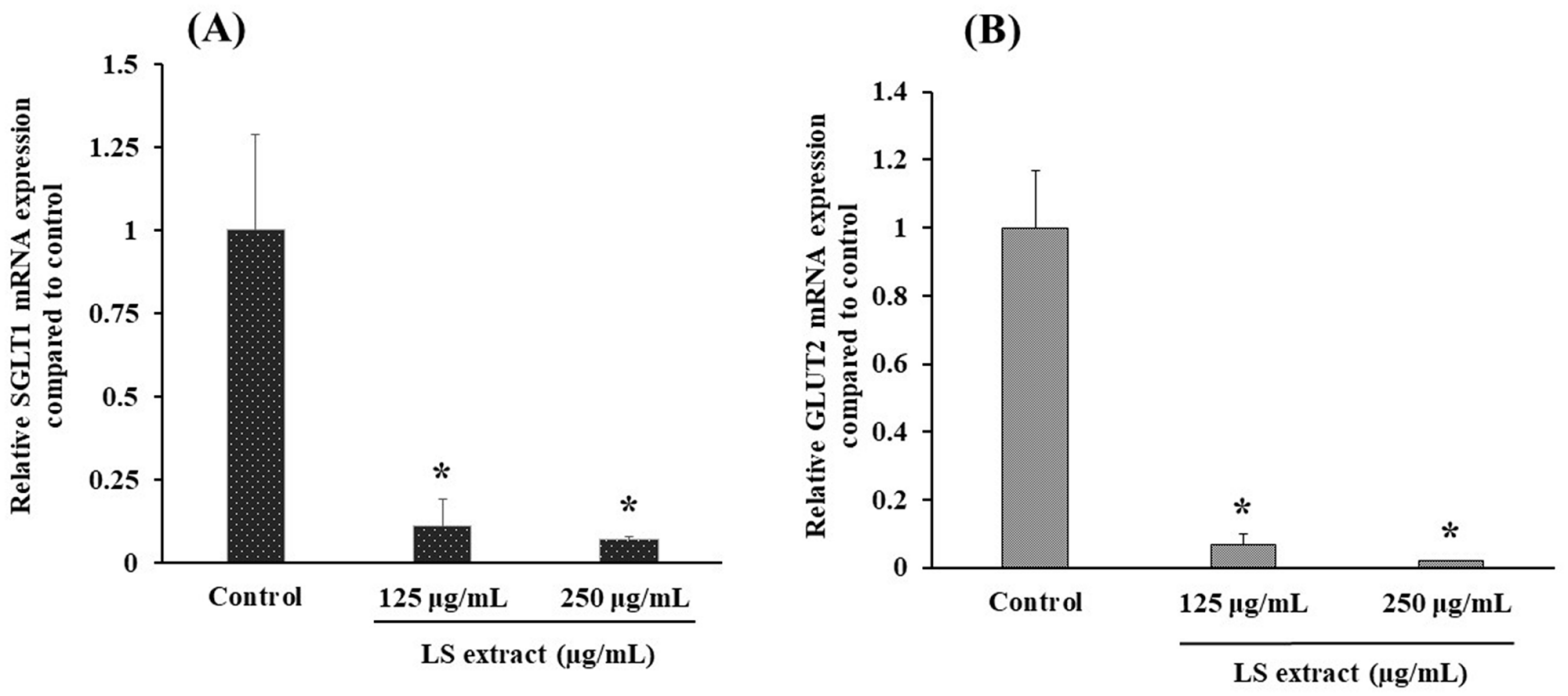
Effect of the LS extract on SGLT1 (A) and GLUT2 (B) mRNA expression. Data are expressed as mean ± *SD* (*n* = 3). *Significant difference compared to control (*p* < 0.05).

Figure 3(A,B) demonstrate the effects of the LS extract on mRNA expressions of glucose transporters in Caco-2 cells. The SGLT1 and GLUT2 mRNA expressions were significantly downregulated by the incubation with the LS extract (125 and 250 µg/mL) (*p* < 0.05).

#### α-Glucosidase activity

**Figure 4.**
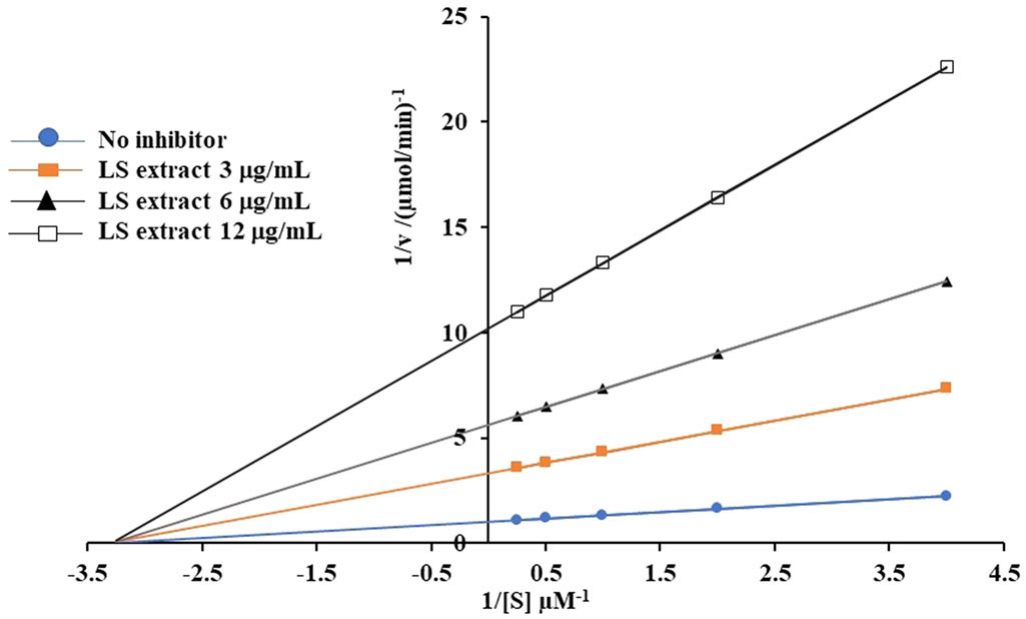
Lineweaver-Burk plot analysis for the kinetic analysis of α-glucosidase inhibitory activity of the LS extract. 1/[*S*] and 1/*v* were the reciprocal of substrate concentration (mM)^−1^ and reciprocal of reaction velocity (nmol/min)^−1^, respectively.

The LS extract exhibited a strong α-glucosidase inhibitory activity in a concentration-dependent manner which was greater than that of acarbose (as a positive control) with the IC_50_ value of 6.52 ± 0.42 and 481.6 ± 26.9 μg/mL, respectively, and statistical differences among the five concentrations employed. An enzyme kinetic analysis from Lineweaver-Burk plots demonstrated that the LS extract inhibited α-glucosidase activity in a non-competitive manner (Figure 4). Kinetic parameters, *V_max_*, *K_m_* and *K_i_*, were calculated to be 0.99 nmol/min, 0.30 mM and 1.32 μg/mL, respectively.

### In vivo *antihyperglycemic activity*

#### OGTT in normal and diabetic mice

**Figure 5.**
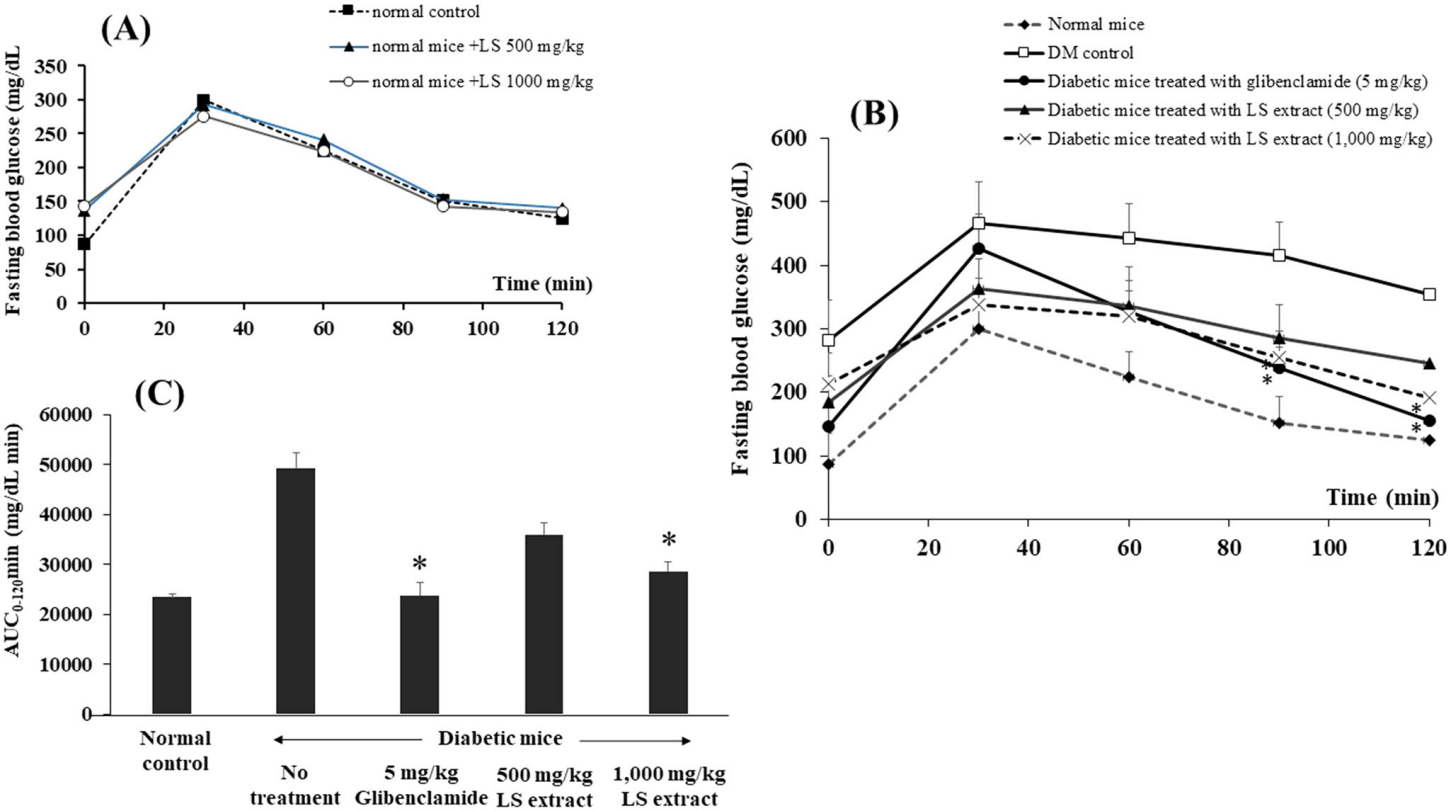
Effects of the LS extract (500 and 1000 mg/kg) on fasting blood glucose levels of normal mice (A) and STZ-NA-induced DM mice (B) and area under the curve (AUC) in oral glucose tolerance test. *Significant difference compared to untreated diabetic control group at correspondent time point (*p* < 0.05).

The FBG concentrations at each time point after oral administration of glucose (0–120 min) in normal and diabetic mice are shown in Figure 5(A,B), respectively. In normal control mice administered with water, OGTT produced an elevation of the blood glucose which peaked at 30 min and gradually returned to baseline within 120 min (Figure 5(A)) with AUC_0–120 min_ of 24,780 ± 300 mg/dL × min. AUC_0–120 min_ of the normal mice treated with the LS extract 500 and 1000 mg/kg were comparable as the normal control group (23,418 ± 315 and 23,436 ± 235 mg/dL· min, respectively).

As presented in Figure 5(B), treatments with glibenclamide (5 mg/kg) and the LS extract (1000 mg/kg) significantly lowered the FBG at 90 and 120 min when compared to untreated diabetic group (*p* < 0.05). Comparison among the AUC_0–120 min_ (Figure 5(C)), it was found that oral administration of the LS extract (1000 mg/kg) exhibited a hypoglycemic effect to a similar extent with glibenclamide. However, an oral treatment with 500 mg/kg LS extract did not produce a marked change in both FBG and AUC_0–120 min_ as compared with DM control group.

### Hypoglycemic effect in STZ-NA-induced diabetic mice after continuous administration of the LS extract

#### Bodyweight and food intake

**Figure 6.**
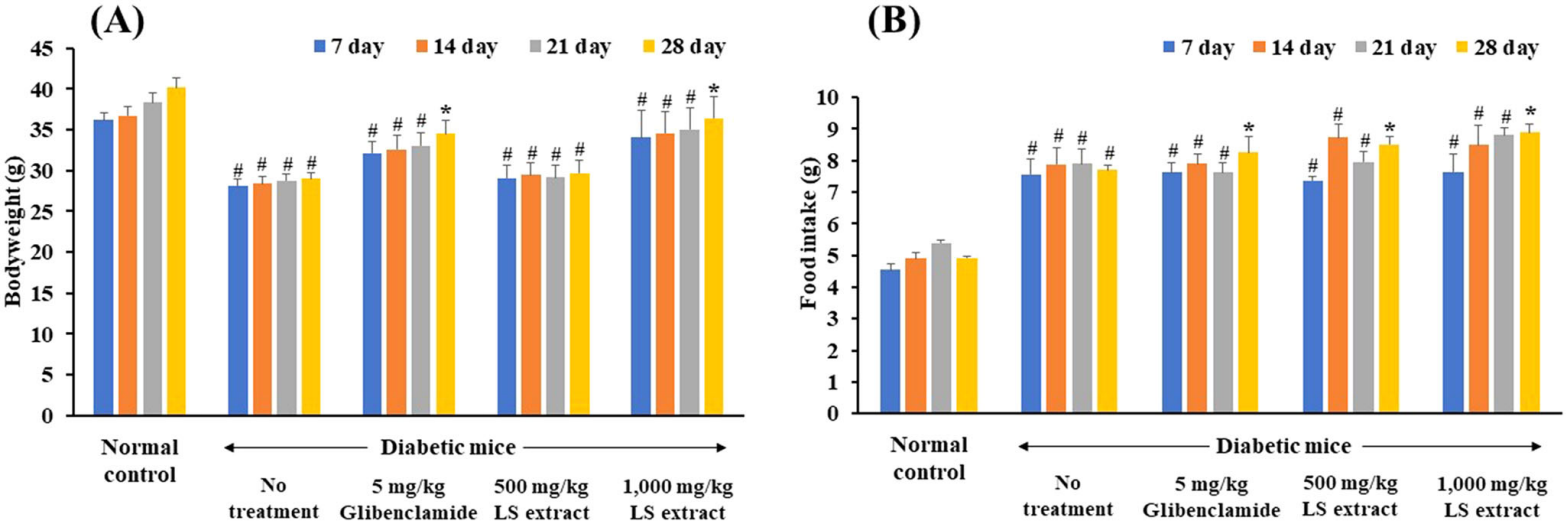
Effect of the LS extract on body weight (A) and food intake (B). Data are expressed as mean ± S.E.M. (*n* = 6–8). ^#^Significant difference compared to normal control group (*p* < 0.05). *Significant difference compared to diabetic control group (*p* < 0.05).

In diabetic mice, mice developed hyperglycemia in day 14–21 after STZ and NA injection. The bodyweights of the normal control mice continuously gained weight while diabetic mice without treatment exhibited a significant weight loss (Figure 6(A)). Continuous administration for 28 days of glibenclamide (5 mg/kg) and the LS extract (1000 mg/kg) significantly increased bodyweight and average food intake as compared with baseline (*p* < 0.05) (Figure 6(A,B)).

#### FBG concentrations

**Figure 7.**
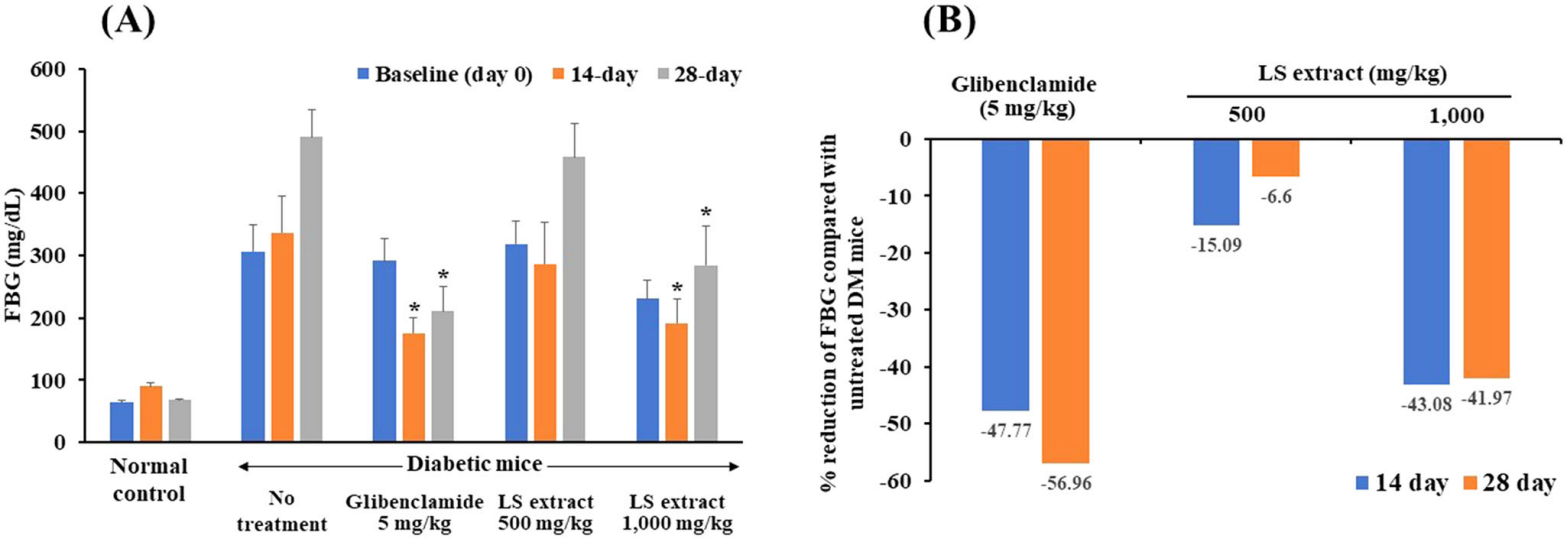
Effect of the LS extract on fasting blood glucose levels (A) and the percentage reduction of fasting blood glucose (B). Data are expressed as mean ± S.E.M. (*n* = 6–8). ^#^Significant difference compared to normal control group (*p* < 0.05). *Significant difference compared to diabetic control group (*p* < 0.05).

The concentrations of FBG of each treatment group are presented in Figure 7(A). As shown in Figure 7(B), after daily administration of 1000 mg/kg LS extract for 14 and 28 days, the FBG concentrations of STZ-NA-induced DM mice were markedly decreased by approximately 43% compared with that of the untreated-diabetic mice (*p* < 0.05). The hypoglycemic effect of the LS extract (1000 mg/kg) was comparable with that of glibenclamide which reduced blood glucose by approximately 47.8% and 57.0% after 14 and 28 days, respectively. However, FBG levels of DM mice treated with 500 mg/kg LS extract did not differ from DM control mice.

#### GLUT4 expression in muscle

As shown in Table 1, GLUT4 expression in muscle was markedly lowered in STZ-NA-induced DM mice as compared with normal control mice (*p* < 0.05). After 28-day continuous treatment with the LS extract (1000 mg/kg) and glibenclamide (5 mg/kg) in STZ-NA-induced DM mice, GLUT4 expression level was significantly enhanced as compared with that of untreated-STZ-NA-induced DM mice (*p* < 0.05). However, the treatment with 500 mg/kg LS extract did not change the GLUT4 expression level.

**Table 1.** Effect of the LS extract on muscle GLUT4, serum insulin, HOMA-IR and oxidative stress markers.

Groups	Muscle GLUT4 (ng/mg protein)	Serum insulin (μIU/mL)	HOMA-IR	SOD1 (ng/mg protein)	MDA (μg/mg protein)	
					Muscle	Pancreas
Normal control	14.33 ± 1.26	6.51 ± 0.76	1.07 ± 0.11	0.17 ± 0.02	0.23 ± 0.03	0.31 ± 0.09
Diabetic control	3.27 ± 0.56^#^	2.40 ± 0.23^#^	4.13 ± 0.63^#^	0.86 ± 0.04^#^	0.46 ± 0.05^#^	0.69 ± 0.08^#^
Diabetic mice treated with glibenclamide (5 mg/kg)	7.00 ± 0.53*	4.86 ± 0.71*	2.20 ± 0.35*	0.57 ± 0.07*	0.31 ± 0.01*	0.34 ± 0.08*
Diabetic mice treated with LS extract (500 mg/kg)	3.35 ± 0.91	2.72 ± 0.32	3.31 ± 0.32	0.81 ± 0.07	0.26 ± 0.03*	0.46 ± 0.07
Diabetic mice treated with LS extract (1000 mg/kg)	8.31 ± 0.72*	4.78 ± 0.37*	2.61 ± 0.28*	0.54 ± 0.09*	0.24 ± 0.02*	0.32 ± 0.06*

GLUT: glucose transporter; HOMA-IR: homeostasis model assessment of insulin resistance; SOD: superoxide dismutase; MDA: malondialdehyde; Data are expressed as mean ± S.E.M. (*n* = 6–8); ^#^significant difference compared to normal control group (*p* < 0.05); *significant difference compared to diabetic control group (*p* < 0.05).

#### Serum insulin and HOMA-IR

After 28-day treatment, untreated STZ-NA-induced DM mice had a significant reduction of serum insulin level as compared with normal control group (*p* < 0.05) (Table 1). Serum insulin levels of the LS extract-treated group (1000 mg/kg) and glibenclamide were markedly increased as compared with that of untreated STZ-NA-induced DM mice (*p* < 0.05).

As summarized in Table 1, HOMA-IR was significantly increased in STZ-NA-induced DM mice as compared with normal control mice (*p* < 0.05). Treatment with 1000 mg/kg LS extract significantly decreased the HOMA-IR level as compared with untreated DM group (*p* < 0.05) and its effect was not significantly different from that of glibenclamide-treated group. Meanwhile, treatment with 500 mg/kg LS extract showed no significant effect to reduce HOMA-IR as compared with untreated DM mice.

### Antioxidant effects

#### *MDA production* in vitro

**Figure 8.**
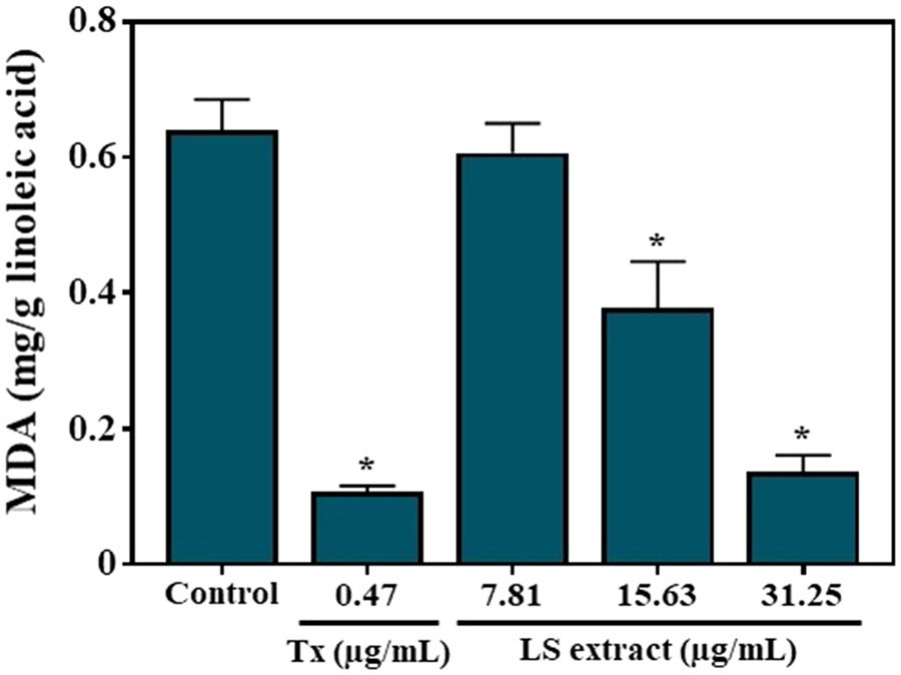
Effect of the LS extract on MDA production in linoleic acid. Data are expressed as mean ± SD (n = 3). *Significant difference compared to control (*p* < 0.05).

The LS extract exhibited an inhibitory effect on the MDA production in a concentration-dependent manner (Figure 8), with statistical differences among the three doses employed, with the IC_50_ value of 24.80 ± 7.24 µg/mL while that of Trolox, as a positive control, was 16.42 ± 1.19 ng/mL.

#### SOD1 in muscle homogenates and MDA in muscle and pancreas homogenates

As shown in Table 1, MDA and SOD1 levels in diabetic control mice extensively increased as compared with normal control mice. After 28-day treatment of DM mice with the LS extract at 1000 mg/kg, SOD1 and MDA levels in muscle and pancreas significantly decreased as compared with untreated DM mice and the antioxidant activity of the LS extract was generally in the same extent as glibenclamide (*p* < 0.05). Administration of the LS extract at 500 mg/kg significantly reduced the MDA levels in muscle as compared with negative control group (*p* < 0.05), whereas it had no significant effects in other tissue homogenates.

## Discussion

Several natural products have been proposed as alternative medicines for the treatment of diabetes. The aqueous LS extract contains several phytochemical active compounds. It has been clinically used as a detoxifying agent for alcohol poisoning (Luengthong et al. 2016; Sukprasert, Deenonpoe et al. 2020). In this study, consistent with the results of an acute toxicity study that reported no gross physical or behavioral changes of mice when treated with LS extract at doses up to 6000 mg/kg for 24 h and 4 weeks (Chutoam et al. 2015), apparent toxicities were not observed in mice treated with either 500 or 1000 mg/kg LS extract.

The possible mechanisms involved in the antihyperglycemic effect of LS leaf extract were investigated at the level of enzymes and transporters. Several active compounds have been observed in the LS leaf extract. As compared with authentic standards, three active compounds, including trilobatin, yanangdaengin, and gallic acid, have been detected in the water extract of LS leaves, consistent with previous studies (Sato et al. 2019; Kongkiatpaiboon et al. 2020). The hypoglycemic and antioxidant effects of the LS extract may be attributed to the integrated effect of its phytochemical active compounds.

The STZ-NA model of T2DM, employed in this study, has been established as an animal diabetic model. Consistently, the present *in vivo* study found that after induction with STZ and NA, the mice significantly developed stable hyperglycemia, polyuria, polydipsia, and weight loss, even though the food intake was increased compared with that of normal mice. These physical and biochemical evidences indicated a successful induction of diabetes in experimental mice. Insufficient insulin levels in T2DM cause a lack of glucose to produce energy, then body starts to metabolize fat and protein in the muscle for energy demands, causing a reduction in overall bodyweight. This lack of energy may cause an increase in hunger, then stimulate appetite, and subsequently increase food intake. Differently from untreated STZ-NA-induced DM mice, a continuous treatment with the LS extract for 28-day improved bodyweight which was similar to that of glibenclamide.

Postprandial hyperglycemia is one of the main causes of diabetic complication. Suppression of glucose production from carbohydrates by the inhibition of α-glucosidase and α-amylase, and inhibition of intestinal glucose uptake are the potential strategies to inhibit glucose absorption in the small intestine (Duarte et al. 2020). To elucidate the effect of the LS extract on postprandial hyperglycemia, an OGTT model using STZ-NA-induced DM mice was employed. After pre-treatment with the LS extract at 1000 mg/kg (p.o.) for 30 min before 2 g/kg glucose loading in STZ-NA-induced DM mice, LS extract substantially retarded the elevation of blood glucose concentrations after glucose loading as compared with that of the control group. According to our *in vitro* results, the LS extract inhibited α-glucosidase activity with an IC_50_ value of 6.52 ± 0.42 μg/mL. The Lineweaver-Burk plots revealed that the LS extract inhibited α-glucosidase by a non-competitive mechanism with a *K_i_* value of 1.32 µg/mL, which is in accordance with a previous kinetic analysis, indicating that trilobatin reversibly inhibited α-glucosidase in a non-competitive manner (He et al. 2022). This effect was stronger than that of acarbose, as positive control. However, our preliminary study found that it has no effect on α-amylase activity (data not shown). These results were corroborated by Noonong et al. (2022) who found that the isolated polyphenols from ethanol extract of LS stems exhibited more potent inhibitory effect towards α-glucosidase than that of α-amylase.

Under regular physiological conditions, glucose resulted from carbohydrate digestion is transferred into the bloodstream through the glucose transporters, SGLT1 and GLUT2, at the enterocytes of the small intestine (Ait-Omar et al. 2011; Röder et al. 2014; Schreck and Melzig 2018). It has been found that the expression of these transporters at the enterocyte membranes is 3-4 fold higher in diabetic patients and consequently worsen T2DM condition due to high glucose surges (Ait-Omar et al. 2011). Therefore, the inhibitions of glucose transport and glucose absorption are the essential strategies for controlling glucose levels in postprandial hyperglycemia (Ait-Omar et al. 2011; Röder et al. 2014). The utilization of Caco-2 cells, the human colonic adenocarcinoma, is one of the well-established models to study the transport mechanisms *in vitro*, because these cells are derived from the enterocytes of small intestine and express the intestinal transporters, i.e., SGLT1 and GLUT2 which are located at the apical and basolateral membranes of the enterocytes, respectively (Röder et al. 2014; Schreck and Melzig 2018). Inhibition of glucose uptake in the intestine through SGLT1 or GLUT2 has currently been used as a strategy to control postprandial blood glucose levels.

In the glucose uptake experiment, incubation of Caco-2 cells with the LS extract for 50 min caused the inhibition of glucose transporters leading to reduction of glucose uptake in Caco-2 cells under a sodium-dependent condition. Furthermore, after incubating Caco-2 cells with the LS extract for 16 h, the mRNA expression of SGLT1 and GLUT2 was decreased. These results indicate that the LS extract exhibits an inhibitory effect on glucose transporters by modulating these transporters at the genomic level.

In general, the mRNA expression of SGLT1 and GLUT2 is regulated by the activation of protein kinase C (PKC) through depolarization-induced Ca^2+^ entry in the enterocytes, ultimately leading to the upregulation of glucose transporter genes (Zheng et al. 2012). Moreover, activation of the AMP-activated protein kinase (AMPK) signaling pathway during energy depletion can also be attributed to the upregulation of SGLT1 and GLUT2 mRNA expression (Kellett and Brot-Laroche 2005; Zheng et al. 2012). Therefore, the LS extract may modulate the activation of PKC and AMPK downstream signaling pathways, which in turn downregulate SGLT1 and GLUT2 mRNA expression.

Medicinal plant-derived polyphenols have been reported to be potential substances involved in the modulation of glucose transporters at the protein and genomic levels in Caco-2 cells (Schreck and Melzig 2018). Johnston et al. (2005) found that polyphenol-glycosides and aglycones blocked SGLT1 and GLUT2, respectively, in Caco-2 cells, resulting in the inhibition of glucose uptake. Phloridzin, a dihydrochalcone type of flavonoid-*o*-glycosides, was used as a positive control in this glucose uptake study, because of its well-known activity as a specific inhibitor of SGLT1 that competitively targets glucose and aglycone-binding sites (Schreck and Melzig 2018). Trilobatin, a major marker of the LS leaf extract, is a positional isomer of phloridzin which is structurally derived from the dihydrochalcone, phloretin (Wang et al. 2020). In a molecular docking study, these two isomers were recently discovered to share the same binding sites on SGLT1 and SGLT2 (Wang et al. 2019). Furthermore, gallic acid has been demonstrated to inhibit the sodium-dependent glucose uptake in Caco-2 cells (Adefegha et al. 2015; Wang et al. 2021). In support of our findings, the polyphenols isolated from LS stem extract reportedly displayed a high binding affinity for SGLT1 and GLUT2 based on molecular docking study, which may prevent glucose from binding to those transporters. Moreover, these polyphenols also downregulated SGLT1 and GLUT2 mRNA expression in Caco-2 cells (Noonong et al. 2022). Therefore, trilobatin, gallic acid, and other polyphenols may contribute to the hypoglycemic effects of the LS leaf extract.

Taken together, these results suggest that the LS leaf extract improved postprandial hyperglycemia through multiple mechanisms of action, including inhibition of glucose production *via* inhibition of α-glucosidase, direct inhibition of intestinal glucose uptake and downregulation of SGLT1 and GLUT2 mRNA expressions.

It is worth noting that the LS extract had no hypoglycemic effect in normoglycemic mice in the OGTT model, whereas it improved the OGTT results in diabetic mice. The exact explanation for this phenomenon has not been clearly elucidated in this study. An increase in GLUT2 expression at the apical side of the brush border membranes in renal and intestinal enterocytes has been reported in animal models of T2DM, as well as in patients with DM (Ait-Omar et al. 2011). Previous reports have also demonstrated the raising of GLUT2 levels at the brush border membranes of intestine, in response to high glucose concentration in the luminal (Kellett and Brot-Laroche 2005). Therefore, inhibition of glucose uptake may be more pronounced under hyperglycemic condition in DM mice than that in normoglycemic mice. In any case, the absence of hypoglycemic effect in normoglycemic condition may be a preferable feature of natural products.

**Figure 9.**
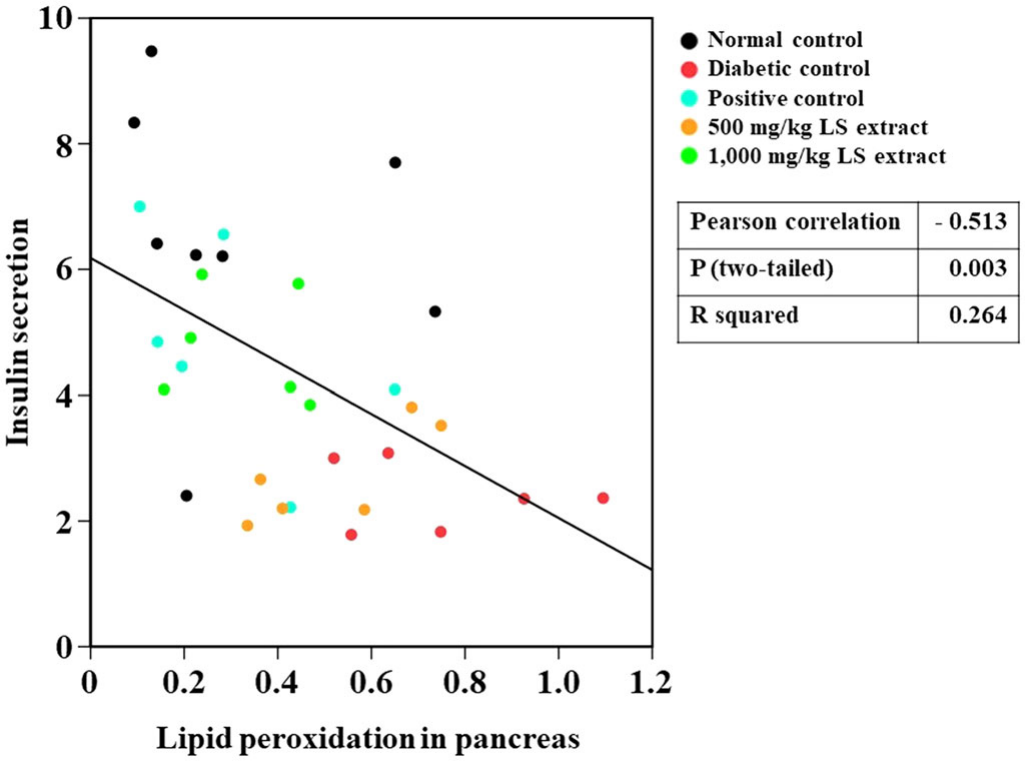
Pearson correlation between lipid peroxidation and insulin secretion (*n* = 32–37).

In this study, a negative correlation was detected between lipid peroxidation and insulin secretion *in vivo* (Figure 9), possibly because insulin secretion is reduced as a consequence of pancreatic β-cells damages owing to increased lipid peroxidation. In accordance with a previous study (Taha et al. 2018), serum insulin level was decreased and MDA concentration was increased in the pancreas and muscle in STZ-NA-induced DM mice compared with those of normal control. However, serum insulin levels were elevated in STZ-NA-induced DM mice after oral treatment with 1000 mg/kg LS extract. Although the antioxidant effect of LS extracts at ROS inhibition steps has been verified in several studies by various *in vitro* models (Maitree et al. 2018; Sato et al. 2019; Kongkiatpaiboon et al. 2020), our study revealed the potent inhibitory effect of LS extract on the final state of lipid peroxidation as a consequence of ROS-induced oxidative stress. In fact, phenolic compounds consist of aromatic ring structures and hydroxyl groups, which can act as reducing agents by donating hydrogen atoms to ROS and further turn them into more neutralized radicals (Iqbal et al. 2015). Thus, the antioxidant effect of LS extract in reducing MDA production may be attributed to its hydrogen-donating capability derived from its phenolic compounds, which leads to the prevention of ROS generation in the early stages of lipid peroxidation. In accompany with the radical scavenging activities of LS extracts as demonstrated in both *in vitro* and *in vivo* studies, therefore, the LS extract may restore pancreatic β-cell functions by ameliorating oxidative stress and subsequently improving insulin secretion. However, further mechanistic approaches toward the direct effect of the LS extract on the regulation of insulin secretion by the pancreas are suggested.

Insulin mediates the mobility of the glucose transporter, GLUT4, to facilitate cellular glucose uptake. After insulin binds to the tyrosine kinase receptor, it induces the activation of protein-kinase B (Akt) and protein kinase C to accelerate the translocation of GLUT4 from the intracellular storage compartments to the plasma membrane (Bryant et al. 2002). Our study found that daily administration of the LS extract (1000 mg/kg) to STZ-NA-induced DM mice for 28 days decreased FBG by approximately 43%, and markedly enhanced serum insulin and GLUT4 concentrations compared to those in untreated DM mice. Therefore, the LS extract may boost insulin production and stimulate the mobilization of GLUT4 to the membranes of skeletal muscle cells to facilitate glucose uptake for energy utilization.

**Figure 10.**
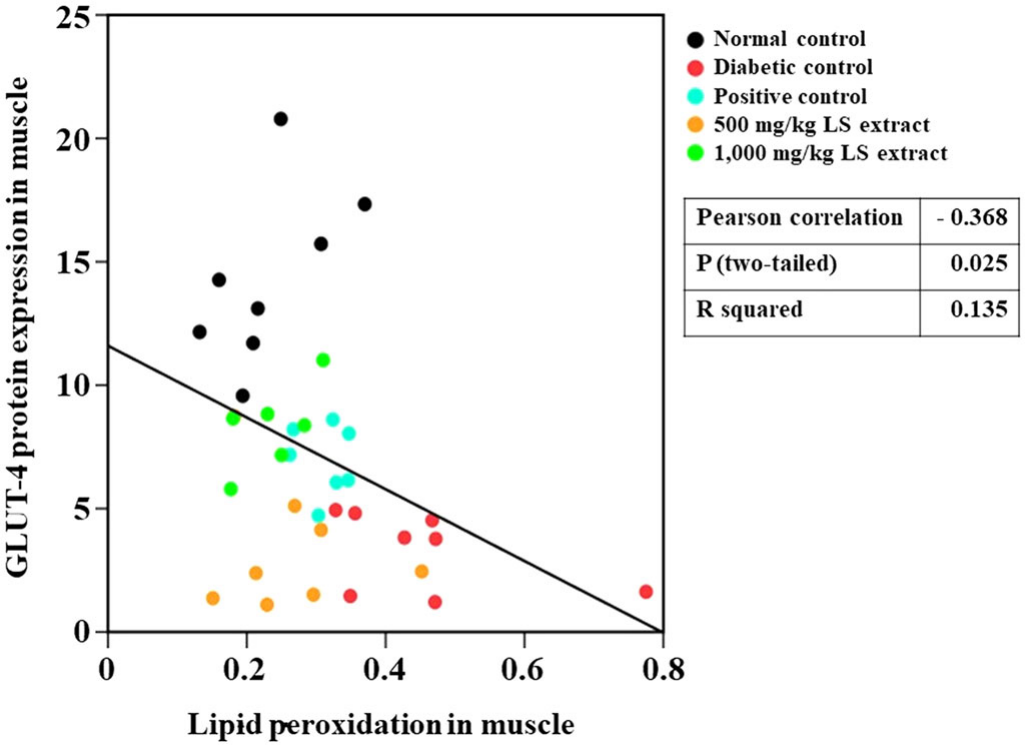
Pearson correlation between lipid peroxidation and GLUT4 levels (*n* = 32–37).

The insulin resistance score, HOMA-IR, has been widely used to evaluate insulin resistance and β-cell function (Onishi et al. 2010). Higher HOMA-IR values indicate impaired insulin resistance. Our results (Table 1) showed that untreated STZ-NA-induced DM mice exhibited substantially higher HOMA-IR values in parallel with an increase in oxidative stress marker levels and reduced GLUT4 protein levels in the muscle, which indicates that insulin resistance was successfully developed in STZ-NA-induced DM mice. We also observed a negative correlation between increased lipid peroxidation and reduced GLUT4 protein levels in the muscles of mice (Figure 10). By the calculation of FBG and serum insulin levels, treatment with the LS extract (1000 mg/kg) remarkably attenuated HOMA-IR as compared with that of the DM control, and its effect was similar to that of glibenclamide, indicating the potent effect of the LS extract in reducing insulin resistance. Moreover, decreases in MDA and SOD1 levels were observed in the LS extract-treated group demonstrating that the LS extract might circumstantially lower the SOD protein in the muscle of diabetic mice by relying upon its antioxidant defense system to eradicate ROS, which eventually improves insulin resistance and promotes GLUT4 translocation.

Trilobatin and gallic acid, the major active components of the LS leaf extract, may contribute to the enhancement of insulin secretion and GLUT4 translocation in the cell membranes, as well as the stimulation of glucose uptake into the cells, because they ameliorate insulin resistance through the insulin receptor substrate-1/phosphoinositide3-kinase/protein-kinase B (IRS1/PI3K/Akt) signaling pathways in the skeletal muscle of STZ-high fat diet-induced diabetic rats (Gandhi et al. 2014; Adefegha et al. 2015; Variya et al. 2020). Therefore, the LS extract may serve to eradicate ROS, promote GLUT4 translocation and eventually improve insulin resistance.

There are some limitations of this study. First, we have not conducted a histological examination of the pancreas to support the improvement in insulin production. Second, we have not examined the possible biochemical pathways involved, such as the IRS1/PI3K/Akt and AMP-activated protein kinase/acetyl-CoA carboxylase (AMPK/ACC) signaling pathways. However, this study is the first to demonstrate *in vitro* and *in vivo* evidences for the antihyperglycemic effects of the aqueous leaf extract of LS, and more detailed research is underway in our laboratory.

## Conclusions

The present study is the first to demonstrate the antihyperglycemic effects of aqueous LS leaf extract by the combined hypoglycemic and antioxidant mechanisms, which include inhibition of α-glucosidase and SGLT1/GLUT2 transporter activities, the elevation of insulin levels and GLUT4 expression in the muscle, and alleviation of oxidative stress (SOD1 and MDA).
